# Evidence for the progression through S-phase in the ectopic cell cycle re-entry of neurons in Alzheimer disease

**DOI:** 10.18632/aging.100044

**Published:** 2009-04-23

**Authors:** David J. Bonda, Teresa A. Evans, Corrado Santocanale, Jesús Catalá Llosá, Jose Viňa, Vladan P. Bajic, Rudy J. Castellani, Sandra L. Siedlak, George Perry, Mark A. Smith, Hyoung-gon Lee

**Affiliations:** ^1^Department of Pathology, Case Western Reserve University, Cleveland, OH 44106, USA; ^2^National Centre for Biomedical Engineering and Science, National University of Ireland Galway, Galway, Ireland; ^3^Departamento de Fisiología, Facultad de Medicina, Avda. Blasco Ibáñez 15, 46010 Valencia, Spain; ^4^Institute of Biomedical Research, Galenika a.d., 11000 Belgrade, Serbia; ^5^Department of Pathology, University of Maryland, Baltimore, MD 21250, USA; ^6^College of Sciences, University of Texas at San Antonio, San Antonio, TX 78249, USA

**Keywords:** Alzheimer disease, cell cycle, DNA replication, minichromosome maintenance protein, neurodegeneration

## Abstract

Aberrant neuronal re-entry into the cell cycle is emerging as a potential pathological mechanism in Alzheimer disease (AD). However, while cyclins, cyclin dependent kinases (CDKs), and other mitotic factors are ectopically expressed in neurons, many of these proteins are also involved in other pathological and physiological processes, generating continued debate on whether such markers are truly indicative of a bona fide cell cycle process. To address this issue, here we analyzed one of the minichromosome maintenance (Mcm) proteins that plays a role in DNA replication and becomes phosphorylated by the S-phase promoting CDKs and Cdc7 during DNA synthesis. We found phosphorylated Mcm2 (pMcm2) markedly associated with neurofibrillary tangles, neuropil threads, and dystrophic neurites in AD but not in aged-matched controls. These data not only provide further evidence for cell cycle aberrations in AD, but the cytoplasmic, rather than nuclear, localization of pMcm2 suggests an abnormal cellular distribution of this important replication factor in AD that may explain resultant cell cycle stasis and consequent neuronal degeneration.

## Introduction

Alzheimer disease (AD) is a progressive and fatal neurodegenerative disease that is clinically characterized by  dementia  and  neurobehavioral  deterioration [1-4].

While the hallmark features of amyloid plaques, neurofibrillary tangles (NFTs), and neuronal loss are well established, the cause(s) of the disease remain elusive. Nonetheless, one mechanism that is gaining increased prominence is the ectopic re-entry of neurons into the cell cycle [5], which accumulate cyclins, CDKs, and other mitotic factors [6-22]. While neuronal cell cycle re-entry mediates AD-type changes [23] and is linked with cell death [24-27],  a number of unanswered questions remain [28]. For example, it is still unclear whether the presence of various cell cycle markers represent a *bona fide *cell cycle or are they, instead, consequential to other pathological processes (e.g., apoptosis). Also, if representative of cell cycle, it is unclear why neurons do not progress and enter cytokinesis. One fitting hypothesis is that some cells undergo hypermitogenic cell cycle arrest, as an alternative to apoptosis, which would result in cell senescence and survival [29].

The minichromosome maintenance proteins are a eukaryotic family of six distinct protein subtypes (Mcm2-7) that are necessary for DNA replication initiation and progression in the cell cycle [30]. During the G1-phase of the cell cycle, the hexameric Mcm2-7 complex assembles at origins of replication on nuclear DNA [31]. Once in S-phase, the complex is phosphorylated by the Cdc7/Dbf4 kinase and the B-type CDKs, and acting as the DNA helicase initiates DNA replication at origins and allows progression of the replication forks [32-37]. The assembly of the Mcm complex is tightly regulated, can occur only in G1 when the activity of CDKs and Cdc7 is low, and is actively prevented once cells enter S-phase till exit of mitosis when the activity of these kinases is high [38], such that replication only occurs once per cell cycle. Expression of Mcm proteins is restricted to actively cycling cells and is a good proliferation marker [39]. While in budding yeast Mcm2-7 proteins shuttle in and out of the nucleus, human Mcms are generally detected in the nuclear compartment [40,41]. Phosphorylation can occur at multiple sites, however phosphorylation of Mcm2 in two adjacent sites Ser40 and Ser41, carried out in succession by CDKs and Cdc7, strictly correlates with cells undergoing or having terminated DNA synthesis [42]. As such, antisera specific for pSer40/41 Mcm2 phosphorylation provides an excellent marker for the detection of cells in a late stage of the cell cycle.

In this study, we compared Ser40/41 Mcm2 phosphorylation in AD and aged-matched control brain. In AD, phosphorylated Mcm2 localized to the cytoplasm of neurons, and strikingly with the characteristic NFT. These findings further support the notion that neurons in AD re-enter the cell cycle, pass through S-phase by activating the only two essential S-phase promoting kinases, and provide evidence for aberrant localization of an essential DNA replication protein.

## Results

### Phosphorylated Mcm2 protein at a CDK- and Cdc7- dependent site is localized to the cytoplasm of AD neurons and targets neurofibrillary tangles and amyloid plaques

**Figure 1.**
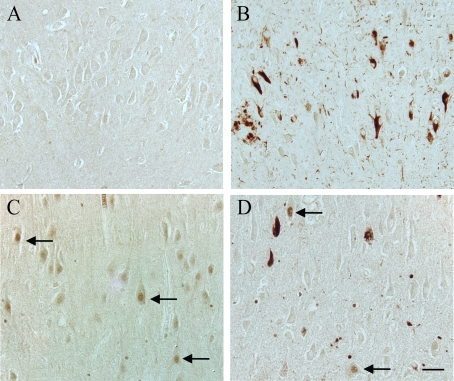
In an 87 year old AD case, hippocampal tissue sections demonstrate significant localization of pMcm2 protein in NFT, dystrophic neurites, and neuropil threads (**B**).  In another AD case, in the CA3 region, in addition to pathological structures, a few pyramidal neuron nuclei (arrows) have significant pMcm2 accumulation (**D**). Most control cases, representative case age 61 years, demonstrate no neuronal staining for pMcm2 protein (**A**), while a few older control cases demonstrate significant nuclear immunolocalization in the pyramidal neurons (control case age 74 years, **C**) Scale bar= 50 μm.

The presence of pSer40/41 Mcm2 (pMcm2) protein was detected using the immunocytochemistry methods discussed in the corresponding section. All of the AD cases examined demonstrated significant accumulation of pMcm2 in NFTs, dystrophic neurites, and neuropil threads (Figure 1B). In most cases, glial nuclei were often stained, and in a small number of cases, some pyramidal cell nuclei within the CA3 region showed significant pMcm2 reactivity (Figure 1D, arrows). In similar areas in most control cases, no staining was seen (Figure 1A), in a small number of aged control cases, pyramidal neuron nuclei showed high pMcm2 protein levels (Figure 1C). In some of the aged controls, a small number of pathological structures (NFT, neuropil threads,etc) were labeled with the pMcm2 antisera (data not shown).

**Figure 2.**
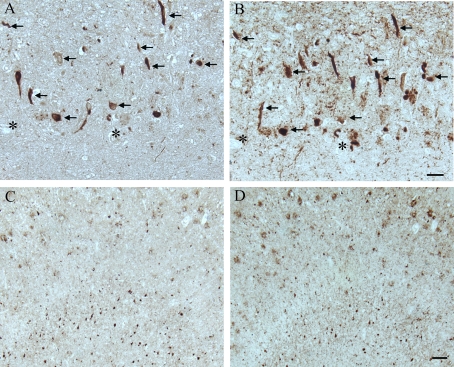
In another AD case, age 63, adjacent hippocampal tissue sections demonstrate many of the AD-related pathological structures (arrows) containing pMcm2 (**A**) are also positive for hyper-phosphorylated tau (**B**) in the CA1 region. Lower magnification of adjacent sections of the subiculum shows the large number of NFT and plaques recognized by pMcm2 (**C**) and AT8 (**D**). * denotes landmark vessel. Scale bars= 50 μm (**A,B**), 100 μm (**C,D**).

**Figure 3.**
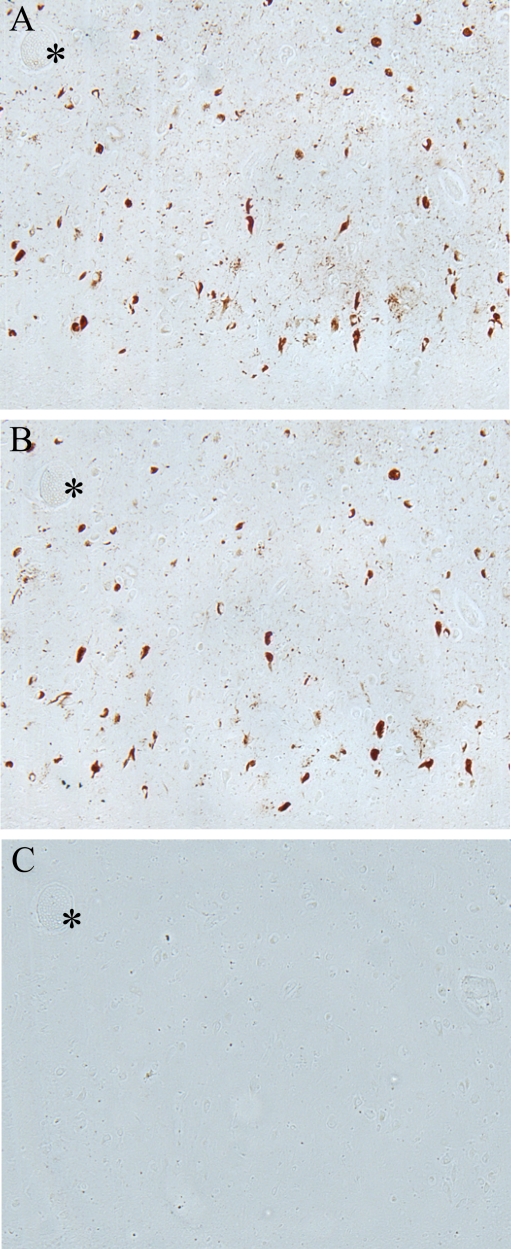
Adsorption of pMcm2 antibody confirms specificity to corresponding pMcm2 antigen. (**A**) AD hippocampal tissue stained with pMcm2 antibody. (**B**) Adjacent section treated with pMcm2 antibody absorbed with non-phosphorylated Mcm peptide demonstrates similar staining. (**C**) Adjacent section treated with pMcm2 antibody absorbed with phosphorylated Mcm2 peptide demonstrates complete absorption. * denotes landmark vessel.

**Figure 4.**
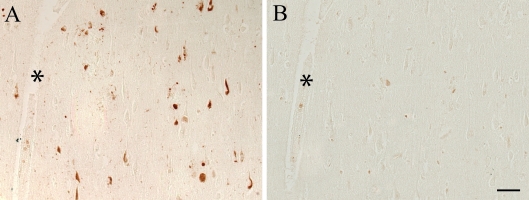
Pretreatment with alkaline phosphatase to remove phosphate groups, results in elimination of pMcm2 reactivity (**B**) compared to an untreated adjacent serial section of an AD case (**A**). * denotes landmark vessel. Scale bar = 50 μm.

All AD cases examined, both with formalin and methacarn fixation, contained many immunoreactive NFT throughout the hippocampus. Additionally, the binding of the anti-pMcm2 antibody to NFT within AD brains was striking and showed some co-localization with phosphorylated tau on adjacent sections of AD tissue In particular, many of the same NFT and senile plaques demonstrated co-localization of tau with pMcm2 in all AD cases (Figure 2). In Figure 3, the specificity of the antibody to pMcm2 protein was confirmed by absorbing antibodies to pMcm2 with phosphorylated  and  non-phosphorylated peptides. As  expected, the phosphorylated peptide completely absorbed the antibody producing no visible staining on the section (Figure 3C) whereas the peptide lacking phosphorylation failed to absorb the antibody (Figure 3B) and produced staining similar to that of the unabsorbed sample (Figure 3A). Further confirmation of the specificity was obtained by treating some sections with alkaline phosphatase to remove phosphate groups. Figure 4 shows that nearly all of the reactivity of the pMcm2 antisera is abolished following dephosphorylation on adjacent sections with (Figure 4B) and without (Figure 4A) alkaline phosphatase pretreatment.

## Discussion

In AD, multiple lines of evidence suggest that neurons vulnerable to degeneration emerge from the post-mitotic, quiescent state and are phenotypically suggestive of cells that are cycling, rather than being in the normal, terminally differentiated, non-dividing state [43]. Such cell cycle re-entry has not only been linked to cell death [44], but has also been implicated in the hallmark pathologies of the disease, namely tau phosphorylation and amyloid-β (Aβ) [23]. Nonetheless, despite the identification of a variety of cell cycle proteins in AD, there remains controversy over whether these are truly indicative of a bona fide reaction of the cell cycle or, instead, reflect the pleotrophic actions of these protein markers [28]. Indeed, proteins previously detected in AD such as Ki67, PCNA, cdc2, cdk4, BRCA1 and pRb [9,44-49], although noted regulators of the mitotic process, are also involved in neuronal processes unrelated to the cell cycle such as DNA repair [50], apoptosis [51], and oxidative stress [52]. Here, however, the detection of a key component of the DNA replication machinery Mcm2, phosphorylated in the Cdk and Cdc7 dependent site Ser40/41 in AD neuronal cytoplasm and NFT not only provides additional support for the cell cycle hypothesis of AD [10], but supports an authentic re-entrant phenotype associated with DNA replication [53]. Mcm2 is in fact not expressed in non-proliferating tissues, as shown in neurons in age-matched control brain, but it accumulates in G1 cells re-entering the cell cycle. Dual phosphorylation of Mcm2 at serine 40 and serine 41, then requires the activity of two kinases whose activity is upregulated in S-phase by the periodic expression of regulatory subunits, Cyclin and Dbf4 [54].

Very intriguingly, pMcm2 in AD neurons, unlike in most cancer cell lines [42], appears to accumulate mostly in the cytoplasm suggesting further degree of deregulation of the MCM complex in disease tissues that may explain the inability of neurons to progress through cytokinesis.

The ectopic re-entry of neurons into the cell cycle likely plays an important role mediating other aspects of AD pathology. Specifically, the microtubule associated protein tau, in cases of AD, exists in a highly phosphorylated form and composes the NFTs that burden the diseased brain, and this increased phosphorylation of tau destabilizes microtubular dynamics and results in neuronal dysfunction [55,56]. Interestingly, while cells are mitotically active, the cell cycle regulator proteins CDKs initiate a similar phosphorylation of tau that precedes the appearance of the NFTs [8] and suggests a possible cause-effect relationship [23]. Similarlythe major protein component of senile plaques is a 4.2 kDa polypeptide termed Aβ, which is derived from a larger precursor (APP) encoded on chromosome 21. Attesting to the importance of this protein, mutations in the APP gene are linked to the inevitable onset of familial AD [57]. Given the probable role of mitotic re-entry in AD, it is notable that APP is upregulated secondary to mitogenic stimulation [58] and that APP metabolism is regulated by cell cycle-dependent changes [59]. Interestingly, Aβ itself is mitogenic *in vitro *[60,61] and therefore may play a direct role in the induction and/or propagation of cell cycle-mediated events in AD. Additionally, Aβ-mediated cell death, at least *in vitro*, is dependent on the presence of various cell cycle-related elements [62]. Most importantly, the ectopic re-entry of neurons into the cell cycle was recently shown to lead to cell death, gliosis, and cognitive deficits—all cardinal features of AD [24].

In conclusion, our results provide further support for the role of cell cycle re-entry in the initiation and progression and AD. As such, cell cycle inhibitors present potential therapies for the disease [63].

## Methods

Tissue. Autopsy tissue samples were obtained using a protocol approved by the Institutional Review Board at University Hospitals of Cleveland. Hippocampal or cortical tissue samples were obtained post mortem from patients (n = 10, ages 63-91 years, mean = 81.8 years) with clinically and histopathologically confirmed AD, as well as from aged-matched controls (n = 8, ages 56-86 years, mean = 70.2 years) with similar post mortem intervals (AD: 2-31 h, mean = 14.5 h; controls: 5-27 h, mean = 15.6 h). All cases were categorized based on clinical and pathological criteria established by CERAD and NIA consensus panel [64]. From the clinical reports available to us, we found no obvious differences in agonal status or other potential confounders between the groups. Tissue was fixed in methacarn (methanol: chloroform: acetic acid; 6: 3: 1 v/v/v) at 4°C overnight or in routine formalin. Following fixation, tissue was dehydrated through ascending ethanol, embedded in paraffin, and 6-μm sections were cut.

Immunohistochemistry. Tissue sections were deparaffinized in xylene, hydrated through descending ethanol, and endogenous peroxidase activity was quenched by 30 minute incubation in 3% hydrogen peroxide in methanol. Non-specific binding sites were blocked with 30 minute incubation in 10% normal goat serum. Sections of both AD and control were immunostained with rabbit polyclonal antibody to Mcm2 phosphorylated at sites Ser40/41 (1:150) [42] or mouse monoclonal antibody to tau (AT8 1:1000) recognizing phosphorylated tau (Ser202/Thr205) (Pierce, Rockford, IL) to identify the location of neuronal pathological structures. Absorption experiments were performed to verify the binding of the Mcm2 Ser40/41 antibody to the appropriate phosphorylated peptide. The primary antibody was incubated in 0.2mg/ml peptide containing 0 or 2 phosphates for 16 hours at 4°C prior to immunostaining. All sections were immunostained using the peroxidase-antiperoxidase with 3-3'-diaminobenzidine as co-substrate as previously described [65].
